# A Composite Score of Insulin Resistance and Inflammation Biomarkers for Predicting Lower Limb Complications in Type 2 Diabetes Mellitus

**DOI:** 10.3390/ijms262411859

**Published:** 2025-12-09

**Authors:** Adina Mitrea, Adela-Gabriela Ștefan, Ionela-Mihaela Vladu, Diana Clenciu, Sorina-Ionelia Stan, Ion-Cristian Efrem, Viorel Biciușcă, Moța Maria, Diana-Cristina Protasiewicz-Timofticiuc, Maria-Magdalena Roșu, Theodora-Claudia Radu-Gheonea, Eugen Moța, Gabriel Mogoș, Delia-Viola Reurean-Pintilei, Lidia Boldeanu, Tiberiu-Ștefăniță Țenea-Cojan

**Affiliations:** 1 Department of Diabetes, Nutrition and Metabolic Diseases, Faculty of Medicine, University of Medicine and Pharmacy of Craiova, 200349 Craiova, Romania; adina.mitrea@umfcv.ro (A.M.); ionela.vladu@umfcv.ro (I.-M.V.); theodora.gheonea@umfcv.ro (T.-C.R.-G.); 2 Department of Diabetes, Nutrition and Metabolic Diseases, Calafat Municipal Hospital, 205200 Calafat, Romania; adela.firanescu@yahoo.com; 3 Department of Internal Medicine-Pneumology, Faculty of Medicine, University of Medicine and Pharmacy of Craiova, 200349 Craiova, Romania; biciuscaviorel@gmail.com; 4 Department of Internal Medicine-Medical Semiology, Faculty of Dentistry, University of Medicine and Pharmacy of Craiova, 200349 Craiova, Romania; cristian.efrem@umfcv.ro; 5 Doctoral School, Faculty of Medicine, University of Medicine and Pharmacy of Craiova, 200349 Craiova, Romania; mmota53@yahoo.com (M.M.); eugenmota@yahoo.com (E.M.); 6 Department of Diabetes, Nutrition and Metabolic Diseases, Faculty of Midwives and Nursing, University of Medicine and Pharmacy of Craiova, 200349 Craiova, Romania; diana.protasiewicz@umfcv.ro (D.-C.P.-T.); maria.rosu@umfcv.ro (M.-M.R.); tiberiu.tenea@umfcv.ro (T.-Ș.Ț.-C.); 7 Department of Surgery, Faculty of Medicine, University of Medicine and Pharmacy of Craiova, 200349 Craiova, Romania; gabrielmogos@yahoo.com; 8 Department of Medical-Surgical and Complementary Sciences, Faculty of Medicine and Biological Sciences, “Stefan cel Mare” University, 720229 Suceava, Romania; delia.pintilei@usm.ro; 9 Consultmed Medical Centre, Department of Diabetes, Nutrition and Metabolic Diseases, 700544 Iasi, Romania; 10 Department of Microbiology, Faculty of Medicine, University of Medicine and Pharmacy of Craiova, 200349 Craiova, Romania; lidia.boldeanu@umfcv.ro

**Keywords:** insulin resistance, inflammation, peripheral arterial disease, diabetic peripheral neuropathy

## Abstract

Diabetes mellitus (DM) is a chronic non-communicable disease associated with macroangiopathy and microangiopathy, with disabling or even life-threatening complications. In the present study, we aimed to analyze the association between insulin resistance (IR) and inflammation biomarkers and peripheral arterial disease (PAD) and diabetic peripheral neuropathy (DPN), respectively. The study had a cross-sectional design and evaluated a panel of IR related indices and inflammatory biomarkers commonly used in clinical and epidemiological research, including the triglyceride-glucose (TyG) index and its obesity related derivates, cholesterol, HDL, glucose (CHG) index, lipid-derived ratios, and composite inflammatory indices, together with interleukin-6 (IL-6), tumor necrosis factor alpha (TNFα) and C-reactive protein (CRP) in 110 subjects, according to the presence or absence of PAD and DPN, respectively. In the PAD (+) group, TyG-waist-to-height-ratio (TyG−WHtR) and CHG recorded significantly increased values (*p* = 0.042, respectively *p* < 0.001), compared to PAD (−). CHG recorded significantly increased values in DPN (+) subjects (*p* = 0.007). In addition, in the PAD (+) subjects, IL-6 and systemic immune inflammation index (SII) recorded significantly increased values (*p* = 0.026, respectively, *p* = 0.015) and TNFα, monocyte to lymphocyte ratio (MLR) and C-reactive protein to albumin ratio (CAR) recorded significantly increased values in DPN (+) subjects (*p* = 0.028, respectively, *p* = 0.008, and *p* = 0.038). We developed a score with a good discriminatory performance for PAD and DPN, including DM duration, TyG−WHtR, SII, MLR and CAR (AUROC 0.822 in PAD, respectively 0.848 in DPN, *p* < 0.001). A composite score combining IR and inflammatory biomarkers showed strong discriminatory performance for lower limb complications in type 2 diabetes, suggesting a valuable tool for early detection and prevention.

## 1. Introduction

Diabetes mellitus (DM) is a chronic non-communicable disease characterized by insulin resistance (IR), insulin deficiency, or both. Globally, the prevalence of DM has reached alarming proportions, estimated at 11.1% [1], and in Romania the Prevalence of Diabetes Mellitus, Prediabetes, Overweight, Obesity, Dyslipidemia, Hyperuricemia, and Chronic Kidney Disease in Romania (PREDATORR) study reported a prevalence of 11.6% [2]. Persistent hyperglycemia can cause chronic macrovascular and microvascular complications, affecting the cardiovascular system, nervous system, kidneys, and retina, with disabling or even life-threatening complications, thus being difficult to manage [1,3,4]. Regular screening for peripheral arterial disease (PAD), foot ulcers, peripheral neuropathy (PN), retinopathy and nephropathy is extremely important, as it prevents both the development and progression of these complications [1].

PAD causes lower limb ischemia, with a significant proportion of patients requiring amputation due to poor prognosis, despite current drug and revascularization treatments [5,6]. Furthermore, PAD is frequently underdiagnosed, because PN may mask ischemic symptoms, such as intermittent claudication or rest pain, leading to an asymptomatic course until advanced stages, when the disease is often detected only through vascular screening or the occurrence of tissue loss [7,8]. This delay emphasizes the need for sensitive and specific diagnostic tools [5]. Vascular inflammation has been associated with numerous biomarkers, such as the erythrocyte sedimentation rate (ESR) [7,9], fibrinogen, C-reactive protein (CRP), and interleukin-6 (IL-6) [7,10,11]. IL-6 levels have been correlated not only with the diagnosis of PAD but also with its severity [7,12]. IR is a hallmark of DM and, like inflammation, is an independent and strongly associated factor with PAD [5,13,14]. A simple and inexpensive indicator of IR is the triglyceride-glucose index (TyG); an increased value of this biomarker is associated with PAD in some studies [5,15]. A recently published meta-analysis demonstrated the association between an increased TyG value and the incidence of arterial stiffness [16].

Diabetic peripheral neuropathy (DPN) is the most common complication of DM, asymptomatic in half of patients, and is often diagnosed at the time of DM detection [17,18]. Patients with DPN are at significant risk of developing ulcers and amputations in the lower limbs [18]. Subclinical inflammation, including certain interleukins (IL), CRP, or tumor necrosis factor-α (TNF-α), seems to play an extremely important role in the development and progression of DPN, an interaction that may lead not only to methods for preventing DM complications but also to the development of new drug therapies [17,19,20]. Persistent inflammation is mediated by numerous effectors, such as the neutrophil-to-lymphocyte ratio (NLR), ESR [17,21,22], the systemic immune-inflammation index (SII) [17,23], or the C-reactive protein-to-albumin ratio (CAR) index [17,24]. IR is associated not only with macrovascular complications but also with microvascular complications. Therefore, TyG is a biomarker that has been correlated with microangiopathy, but limited data are available [3]. To date, a correlation between TyG and type 2 DM and its complications has been demonstrated, and combining TyG with obesity-related indicators is considered to be an effective method to improve its predictive value [25,26].

In this study, we aimed to analyze IR biomarkers such as TyG and its derivatives in combination with obesity-related indicators. Similarly, we aimed to include in the analysis the triglyceride-to-high-density lipoprotein cholesterol (TG/HDL-c), metabolic score for insulin resistance (METS-IR), and the cholesterol, HDL, glucose (CHG) index and its derivatives, since we did not find any data available so far regarding their association with PAD or DPN. Moreover, we aimed to analyze the mentioned inflammatory biomarkers, as well as other markers such as ferritin, fibroblast growth factor 1 (FGF1), matrix metalloproteinase 1 (MMP1), monocyte-to-lymphocyte ratio (MLR), and the neutrophil percentage-to-albumin ratio (NPAR). The aim of the present study was to analyze the association between IR and inflammation biomarkers and PAD and DPN, respectively.

## 2. Results

A total of 160 subjects were evaluated for PAD and DPN. Of the subjects selected for enrollment, 32 patients refused to participate in the study. However, data were incomplete for another 18 participants; so, they could not be included in this study. Thus, a total of 110 subjects were considered eligible for the statistical analysis performed, with the majority of subjects being males, both in the PAD (+) group (51.2%) and in the DPN (+) group (54.3%). The characteristics of the subjects enrolled in the present study including demographic, clinical and biochemical data are presented in Table 1. The distribution of subjects between PAD− and PAD+ and between DPN− and DPN+ reflects the clinical, profile of the hospitalized cohort evaluated for lower limb complications; consequently, more complex multivariable models were not pursued, as they would not have produced stable or reliable estimates in this sample.

In the PAD group, statistical analysis revealed significant differences between PAD (+) and PAD (−) subjects, such that PAD (+) participants presented with older age, a longer duration of DM, and significantly increased values of FPG, HbA1c, TC, and LDL-c. Although there were more men in the PAD (+) group than women, no statistically significant differences were recorded (*p* = 0.231). Moreover, in the PAD (+) group, higher values of BMI, WC, WHtR and HDL-c were recorded, but statistical significance was not reached compared to the PAD (−) group.

Similarly, in the DPN group, the following statistically significant differences were recorded: DPN (+) subjects presented older age, longer duration of DM, increased TC and LDL-c values, compared to DPN (−) subjects. However, statistical significance was not recorded for the other analyzed characteristics.

In Table 2, we calculate and compare the IR indicators according to the presence or absence of PAD and DPN respectively. It was found that in the PAD (+) group, TyG−WHtR and CHG recorded statistically significant differences (*p* = 0.042, respectively, *p* <0.001), having increased values compared to PAD (−) subjects. Regarding DPN, only CHG recorded significantly increased values in DPN (+) subjects, compared to DPN (−) (*p* = 0.007). The other analyzed IR biomarkers did not reach significant differences between the groups.

Similarly, in Table 3, we compare the inflammation indicators according to the presence or absence of PAD and DPN. In the PAD (+) subjects, IL-6 and SII recorded significantly increased values (*p* = 0.026, respectively, *p* = 0.015), compared to PAD (−) group. In addition, TNFα, MLR, and CAR recorded significantly increased values in DPN (+) subjects, compared to DPN (−) (*p* = 0.028, respectively, *p* = 0.008, and *p* = 0.038). The other analyzed inflammation biomarkers did not show statistical differences between groups.

Regarding ROC curve analysis in PAD, the largest AUROC curve was recorded in the case of DM duration, with a value of 0.813, and the cut-off value was 7.50, with 70.7% sensitivity and 78.6% specificity (*p* < 0.001). Among IR indicators, the largest AUROC curve was recorded in the case of TyG−WHtR, with a value of 0.629, and among inflammation indicators, the largest AUROC curve was recorded in the case of SII and IL-6, with a value of 0.655 and 0.641, respectively (Table 4).

Regarding ROC curve analysis in DPN, the largest AUROC curve was also recorded in the case of DM duration, with a value of 0.982, and the cut-off value was 4.50, with 91.3% sensitivity and 100.0% specificity (*p* < 0.001). Among inflammation indicators, the largest AUROC curve was recorded in the case of MLR, with a value of 0.697, followed by TNFα and CAR, with a value of 0.664 and 0.655, respectively (Table 5).

We took into account the cut-off values resulting from the ROC curve analysis for DM duration, as well as for IR and inflammation biomarkers that are easy to calculate and use, associated with PAD and DPN in the present study, to develop a score (Table 6), which contains five items, with 1 point being awarded for each item.

**Figure 1:**
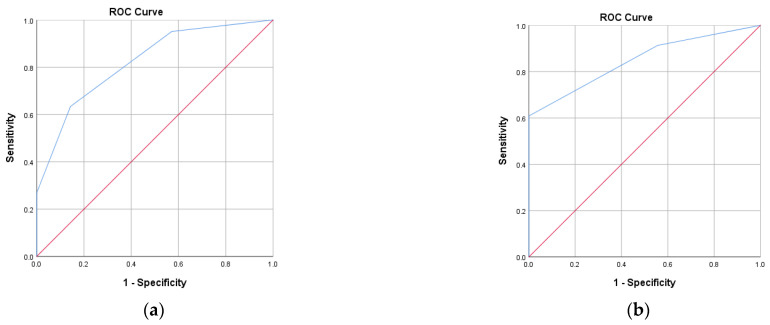
The ROC curve analysis for the proposed score for the detection of lower limb complications (n = 110). (**a**) PAD; (**b**) DPN. Red line—Reference line; Blue line—ROC curve.

The proposed score presented an AUROC of 0.822 in the case of PAD, respectively, 0.848 in the case of DPN, with a cut-off value of 2.50 in both ROC curve analyses, having a statistically significant discriminatory performance (*p* <0.001) in the present study (Table 7) (Figure 1).

## 3. Discussion

In recent years, the prevalence of DM has reached alarming levels, and with it, an epidemic of its macrovascular and microvascular complications has emerged. Understanding the complex interplay between metabolic dysregulation, inflammation, and vascular impairment remains essential for improving the early detection and prevention of diabetes complications, which continue to represent major causes of morbidity, disability, and health-care burden among individuals with type 2 DM. Our findings complement other recent research that addresses different pathways involved in diabetes complications [27], emphasizing the multifactorial nature of diabetic tissue damage and the need for diverse biomarker approaches.

Building on this idea, we assessed insulin resistance using surrogate indices derived from routine biochemical parameters. These markers are widely used in studies investigating metabolic and vascular complications in type 2 DM and have demonstrated reliable performance in reflecting underlying insulin resistance across different populations [28,29,30,31]. Their usefulness has also been supported by recent Romanian data [25,26], reinforcing their applicability in cohorts similar to ours and providing a coherent framework for interpreting the associations observed in this study. Direct methods such as the hyperinsulinemic–euglycemic clamp, although considered the gold standard [32,33], are invasive, resource-intensive, and impractical for routine clinical use or for hospitalized patients undergoing multiple investigations. Likewise, HOMA-IR and HOMA-β were not appropriate for our cohort, as many hospitalized participants were receiving various glucose-lowering therapies—including insulin—which directly influence fasting insulin concentrations and therefore compromise the validity of HOMA-based indices [34,35]. For these reasons, the surrogate IR biomarkers employed in our study are advantageous in clinical practice, as they rely solely on FPG, TC, TG, and HDL-c, which involve minimal costs and are routinely measured. The association between elevated TyG index values and metabolic syndrome or type 2 DM has been demonstrated in our previous studies [26,36], and similar findings have been reported in relation to PAD, where higher TyG values were observed in affected individuals [5,15,37]. In our analysis, however, TyG–WHtR and CHG—two IR-related composite markers whose relationship with PAD has not been examined previously to our knowledge—showed stronger discriminatory performance than the TyG index itself, suggesting that indices integrating lipid–glucose dynamics with anthropometric components may better capture IR-associated vascular impairment. The report by Caliskan and Boyuk [37] identifying TyG as an independent predictor of PAD highlights the importance of considering metabolic factors in this context, although their findings must be interpreted alongside the established contribution of smoking to oxidative stress, endothelial dysfunction, and metabolic abnormalities [38,39]. Because smoking could significantly influence IR-related biomarkers, we excluded smokers from our analysis to minimize confounding and ensure a clearer evaluation of these associations. Another study published by Ning et al. [15] demonstrated a higher value of the TyG index in subjects with PAD and identified TyG as an independent risk factor for this complication (OR = 3.92, *p* < 0.001), further supporting the involvement of IR-related metabolic disturbance in lower-limb atherosclerosis, in line with the findings of Pala et al. [40] and Zhao et al. [41] that demonstrated the predictive value of TyG for the occurrence of chronic limb-threatening ischemia in patients with PAD. By contrast, Miranda et al. [3] did not demonstrate a positive correlation between TyG and macrovascular complications, which was due, according to the authors, to the study small size; their results were consistent with our own findings, in which TyG did not differ significantly between PAD (+) and PAD (−) groups, whereas derived indices such as TyG–WHtR and CHG were more informative. In agreement with a previously published study [15], our results show that subjects with PAD presented with older age compared to subjects without this chronic complication. Moreover, the same publication demonstrates that PAD had a higher incidence among females compared to males, which can be explained by the influence of factors such as metabolic syndrome, obesity, depression, and changes in estrogen secretion [42]. Regarding DPN, a study published in 2023 [43], which analyzed 500 patients, found a significantly higher TyG value in subjects with DPN compared to the control group. In our study, however, no statistically significant differences were found between groups regarding TyG, but only in the case of CHG. According to Kassab et al. [43], a higher TyG value is an independent factor associated with the severity of DPN, the association being more significant in men, aged over 65 and with a DM duration over 10 years. The study published by Miranda et al. [3] demonstrated a significant association between the TyG index and DPN. It also described subjects with DPN as having a longer duration of DM, increased FPG values, and a higher TyG value, compared to the control group; their results were similar to those in our study. However, there were also studies [44,45] that did not report a statistically significant association between the TyG index and DPN.

HbA1c can be used to assess the risk of developing PAD [46]; this association is determined by long-term elevated blood glucose, which leads to the activation of platelets and the protein kinase C pathway, inducing a proinflammatory state and ultimately causing increased oxidative stress, as well as endothelial dysfunction [47]. In our study, HbA1c showed statistically significantly higher values in subjects with PAD, compared to the control group (8.90 vs. 6.60%, *p* = 0.002).

Inflammatory activation is a central mechanism in atherosclerotic disease progression [7,48], and several studies have highlighted the relevance of IL-6 in this context. Studies by Sabat et al. [49] and Zaib et al. [7] identified IL-6 as a predictor of PAD; their results were confirmed by our study (8.71 vs. 7.29 pg/mL, *p* = 0.026). CRP production is stimulated by IL-6 in hepatocytes and adipose tissue, with elevated levels indicating inflammation or tissue injury [7,50]. Thus, CRP and MMP1 concentrations have been shown to be significantly increased in patients with PAD [11,51,52], in disagreement with the results of our study. The importance of lipid–inflammation interplay in vascular disease [53] supports our integrative approach to complication risk prediction in type 2 DM.

A study published in 2016 by Duksal et al. [54] demonstrated significantly increased levels of TNF-α in subjects with type 2 DM and DPN, compared to those without DPN; their results were similar to those in the present study. Another study [55] highlighted significantly increased levels of IL-6 in this category of subjects. In addition, Akintoye et al. [56] reported significantly increased levels of IL-6, as well a significant decrease in NLR in subjects with DPN, compared to the control group; their results are discordant with our findings. In contradiction to Akintoye’s results, Liu et al. [21] reported a significant increase in NLR in subjects with DNP with this biomarker being associated with vibration perception threshold and nerve conduction velocity.

CRP has been shown to be an independent predictor for the presence of DPN, recording significantly increased values in these patients [57]. Another study confirmed the association of NLR, CRP, and ESR with DNP [22]. In a study published by Li et al. [23], an increased SII value was significantly associated with DPN, as well as with a higher vibration perception threshold. These findings are consistent with our results (cut-off value 334.27, with a sensitivity of 87.8% and a specificity of 50.0%). A biomarker recently proposed by Aktas [24], namely CAR, has been shown to have significantly increased values in subjects with DPN, thus being considered an indicator of the risk of developing DPN; their results were similar to those reported in the present study.

In the present study, we proposed a composite score combining both IR and inflammatory biomarkers, which demonstrated high discriminatory performance for both PAD (AUROC 0.822) and DPN (AUROC 0.848). The inclusion of easily measurable laboratory and clinical parameters increases its applicability in daily practice. Furthermore, the composite score proposed in this study was constructed using variables that demonstrated the most consistent and clinically relevant associations with PAD and DPN and that reflected complementary mechanisms, including disease chronicity (DM duration), insulin resistance (TyG–WHtR), and systemic inflammation (SII, MLR, CAR). While additional biomarkers showed statistical significance, they represented overlapping physiological domains or shared computational components with those included in the score and were therefore not retained. In developing the score, we also considered the potential for multicollinearity between indices derived from similar biochemical parameters and avoided combining closely related markers. This integrative approach aligns with current trends emphasizing multimarker risk assessment in diabetes, offering a feasible tool for early identification of patients at risk for lower limb complications.

This study also has limitations, represented by the fact that the study was conducted in a certain geographical area; hence, the results cannot be extrapolated to the general population. The study design is cross-sectional, which is why we could not establish a causal relationship between IR and inflammation biomarkers and the two complications of type 2 DM, PAD and DPN. The evolution of changes in these biomarkers over time would have provided additional data. Furthermore, only hospitalized patients evaluated for symptoms suggestive of lower limb complications were included, which influenced the subgroup composition and may limit broader applicability. This subgroup composition together with the small size of the analyzed sample was not suitable for multivariate logistic regression analysis, but the data obtained by us can be applied to a limited extent, within populations with similar characteristics to the subjects enrolled in the present study. In addition, the differences in age and diabetes duration between subgroups could not be adjusted for through multivariable modeling, and this should be considered when interpreting the associations reported. Regarding the proposed composite score, given the structure of our cohort, which was not appropriate for regression-based weighting, this score is intended as an exploratory framework, as it was derived within a single cohort and was not subjected to internal validation procedures; its performance therefore requires confirmation in independent samples.

Our study also has strengths, one of which is the inclusion of novel markers of IR and inflammation that have not been previously analyzed in association with PAD or DPN. Another strength is that the diagnosis of PAD and DPN was established according to current guidelines and not based on patient-reported symptoms or personal history.

The subject we addressed in this study has not been sufficiently investigated to date. Multicenter studies are needed to validate the results, as well as longitudinal studies, to establish a clear causal relationship between these simple biomarkers and PAD, respectively, DPN, in order to facilitate prevention.

## 4. Materials and Methods

### 4.1. Participants

We conducted a cross-sectional non-interventional study, carried out between February 2023 and July 2023, using The Strengthening the Reporting of Observational Studies in Epidemiology (STROBE) Statement [58]. We calculated the sample size using https://www.calculator.net/ (accessed on 20 February 2023) [59], with a 95% confidence interval (CI), which corresponds to a z-score of 1.96 and a 5% margin of error. We assumed that 25% of patients might refuse to participate in the study, and 10% of subjects might have incomplete data. Therefore, for the sample to be representative, we estimated that 110 subjects should be enrolled in the study.

The inclusion criteria in the study were patients over 18 years of age diagnosed with type 2 DM, who were hospitalized in the Endocrinology and Diabetes Clinic of the Clinical Hospital Filantropia of Craiova, Romania, for symptoms suggestive of lower limb chronic complications who underwent evaluation for the presence of PAD, respectively, DPN. The exclusion criteria were age under 18 years, subjects without DM or diagnosed with prediabetes, type 1 DM, or other forms of DM, conditions that may influence the results of the investigations, such as smoking or alcohol consumption status, as well as patients with serious comorbidities (neoplasia, end-stage liver or kidney disease) and inflammatory diseases or infectious diseases. All study participants signed the informed consent form before undergoing the procedures. The study was conducted in accordance with the World Medical Association Declaration of Helsinki—Ethical Principles for Medical Research Involving Human Participants, and with the applicable standards of the International Conference on Harmonization (ICH)/Good Clinical Practice (GCP) and was approved by the Scientific Ethics and Deontology Committee of the University of Medicine and Pharmacy of Craiova, approval number 46/26 January 2023.

### 4.2. Anamnestic, Socio-Demographic, Clinical, and Biochemical Data

We recorded significant information regarding socio-demographic characteristics (gender, age), but also the medical history of DM.

Anthropometric data such as the weight, height, and waist circumference (WC) were recorded using non-invasive measurements, easy to use in clinical practice. Subsequently, we calculated the body mass index (BMI), using the formula: BMI = weight (kilograms)/height squared (meters). We also calculated the waist-to-height ratio (WHtR) according to the formula WC (cm)/height (cm).

Biochemical data were obtained by collecting venous blood in a vacutainer, each of which was labeled with the identification number of the respective participant. All biomarkers, including glycemic, lipid, IR-derived and inflammatory parameters, were measured from fasting venous blood samples collected in the morning after an overnight fast of at least 8 h. Analyses were performed according to standardized procedures in the laboratory of the Hospital Filantropia Craiova. Enzymatic methods were used to determine the fasting plasma glucose (FPG), total cholesterol (TC), high-density lipoprotein cholesterol (HDL-c), low-density lipoprotein cholesterol (LDL-c), and triglycerides (TG). Immunoassay was used to determine C-reactive protein (CRP) and other biomarkers, such as tumor necrosis factor alpha (TNFα), interleukin-6 (IL-6), fibroblast growth factor 1 (FGF1), and matrix metalloproteinase 1 (MMP1). Turbidimetry was used in order to determine glycated hemoglobin (HbA1c), fibrinogen, and albumin, and the Westergren method was used for erythrocyte sedimentation rate (ESR). The analysis of the blood count was performed using the laser flow cytometry method. Moreover, we calculated certain inflammation biomarkers, using the formulas presented in Table 8.

Regarding the IR component analyzed in the present study, IR biomarkers were calculated according to the formulas presented in Table 9.

### 4.3. Evaluation of Peripheral Arterial Disease and Diabetic Peripheral Neuropathy

To assess PAD, we used the ankle–brachial index (ABI), which is a low-cost and easy-to-use method in clinical practice, demonstrating good concordance with the Doppler method. An ABI value ≤0.90 confirmed the diagnosis of PAD [63].

The Toronto score was used to assess DPN. Subjects with specific symptoms in the lower limbs, diminished or abolished patellar and Achillean reflexes, as well as those with modified sensibility tests (the 10 g monofilament for pressure perception, the TipTherm test for temperature perception, the pricking test with neurotips, the Rydel-Seiffer tuning fork for vibration perception, and proprioception tests) were diagnosed with DPN, according to the criteria: (a) 1 point is awarded for each symptom present; (b) 1 point is awarded for each test applied unsuccessfully, 1 point if reflexes are diminished, and 2 points if reflexes are abolished; (c) if the Toronto score had a score above 6 points, the diagnosis of DPN was established [64].

### 4.4. Statistical Analysis

To identify the distribution of continuous variables, the Kolmogorov-Smirnov test was used. If the tested variables had a Gaussian distribution, they were reported as the mean ± standard deviation (SD), and those with a non-Gaussian distribution were reported as the median and interquartile range (IQR). To determine statistically significant differences between groups, Student’s *t* test was used to compare means, and the Mann–Whitney U test was used to compare medians. The chi-square test was used to analyze categorical variables.

Given the size and structure of the study population, including the imbalance between subgroups, multivariable logistic regression could not be reliably performed. The statistical evaluation was therefore based on comparisons between groups and receiver operating characteristic (ROC) curve analyses. The biomarker comparisons were exploratory in nature, and no formal correction for multiple testing was applied; the study aimed to identify potential patterns that could justify future confirmatory research.

The cut-off values for IR and inflammation biomarkers that demonstrated significant associations with PAD or DPN were determined by analyzing the area under the ROC (AUROC) curve. From these, five variables were selected for inclusion in the proposed composite score (DM duration, TyG–WHtR, SII, MLR, CAR) based on their consistent associations with PAD and DPN and their ability to represent distinct and complementary biological domains. For these five variables, the cut-off thresholds were defined using the Youden index, and the exact values were retained to reflect the optimal discrimination identified in our dataset. Variables sharing the same computational elements or reflecting closely overlapping physiological pathways were not combined to avoid conceptual redundancy. As multivariable logistic regression was not feasible, the proposed composite score was designed as an exploratory clinical tool rather than a regression-derived model. Internal validation or cross-validation was not performed, as the dataset did not allow for reliable partitioning into training and testing subsets.

Results were considered statistically significant if a *p* value < 0.05 was recorded. Data analysis was performed using the Statistical Package for the Social Sciences (SPSS) version 26.0 (SPSS Inc., Chicago, IL, USA).

## 5. Conclusions

PAD and DPN remain among the most frequent and disabling chronic complications in patients with diabetes mellitus. Our study demonstrated that, beyond disease duration, simple and routinely available markers of insulin resistance (TyG–WHtR) and inflammation (SII, MLR, and CAR) are significantly associated with these lower limb complications. Based on these findings, we propose a five-item composite score that integrates both metabolic and inflammatory parameters, providing a practical and inexpensive tool for identifying patients at higher risk for PAD and DPN. This easily applicable model may assist clinicians in early risk stratification and in guiding preventive strategies. Nevertheless, the proposed score requires validation in larger, multicenter, and longitudinal cohorts to confirm its good discriminatory performance and clinical utility.

## Figures and Tables

**Table 1:** Demographic, clinical, and biochemical characteristics of the study participants.

Characteristics	PAD			DPN		
	PAD (−)	PAD (+)	*p* Value	DPN (−)	DPN (+)	*p* Value
Participants, no.	28	82		18	92	
Gender (%) *			0.231			0.925
Males	64.3	51.2		55.6	54.3	
Females	35.7	48.8		44.4	45.7	
DM duration (years) **	4.00 (6.00)	15.00 (15.00)	<0.001	1.00 (2.00)	15.00 (13.00)	<0.001
Age (years) ***	54.14 ± 10.64	63.88 ± 9.20	<0.001	55.56 ± 12.99	62.54 ± 9.55	0.009
BMI (kg/m^2^) **	31.00 (8.00)	32.00 (8.00)	0.474	33.00 (7.00)	32.00 (9.00)	0.163
WC (cm) **	103.50 (10.00)	104.00 (18.00)	0.303	107.00 (25.00)	104.00 (19.00)	0.348
WHtR **	0.60 (0.14)	0.64 (0.13)	0.099	0.65 (0.14)	0.62 (0.11)	0.213
FPG (mg/dL) **	153.00 (131.00)	230.00 (129.00)	0.003	162.00 (197.00)	225.00 (133.00)	0.137
HbA1c (%) **	6.60 (3.00)	8.90 (3.00)	0.002	6.30 (4.20)	8.80 (3.10)	0.195
TC (mg/dL) **	216.50 (59.00)	176.00 (68.00)	0.007	232.00 (139.00)	176.00 (67.00)	0.004
HDL-c (mg/dL) **	36.00 (17.00)	42.00 (16.00)	0.138	36.00 (30.00)	40.50 (17.00)	0.846
LDL-c (mg/dL) **	108.20 (46.20)	87.80 (39.80)	0.004	109.00 (61.00)	92.40 (42.30)	0.018
TG (mg/dL) **	139.50 (364.00)	171.00 (142.00)	0.402	141.00 (502.00)	169.50 (150.00)	0.698

PAD: peripheral artery disease; DPN: diabetic peripheral neuropathy; DM: diabetes mellitus; BMI: body mass index; WC: waist circumference; WHtR: waist-to-height ratio; FPG: fasting plasma glucose; HbA1c: glycated hemoglobin; TC: total cholesterol; HDL-c: high-density lipoprotein cholesterol; LDL-c: low-density lipoprotein cholesterol; TG: triglycerides. Continuous variables with normal distribution are presented as mean ± standard deviation, and those with abnormal distribution are presented as median (IQR). * chi-square test was used for this analysis; ** Student’s *t* test was used for this analysis; *** Mann–Whitney U test was used for this analysis.

**Table 2:** IR biomarkers in patients with PAD vs. DPN.

IR Biomarkers	PAD			DPN		
	PAD (−)	PAD (+)	*p* Value	DPN (−)	DPN (+)	*p* Value
TyG *	9.30 (2.04)	9.73 (1.02)	0.084	9.25 (2.45)	9.71 (1.05)	0.245
TyG−BMI *	304.47 (72.20)	311.51 (81.16)	0.272	306.34 (39.85)	311.20 (90.93)	0.583
TyG−WC *	1013.17 (210.91)	1045.63 (162.67)	0.197	1045.63 (193.52)	1033.74 (189.87)	0.698
TyG−WHtR *	5.94 (1.72)	6.31 (1.28)	0.042	6.14 (1.25)	6.19 (1.37)	0.572
TG/HDL-c *	3.44 (10.83)	4.18 (4.06)	0.989	3.57 (15.19)	4.18 (4.31)	0.859
MetS-IR *	54.89 (10.19)	55.84 (14.92)	0.410	56.45 (6.75)	55.83 (15.04)	0.821
CHG **	6.15 ± 1.01	6.15 ± 0.63	<0.001	6.14 ± 0.63	6.23 ± 1.17	0.007
CHG−BMI *	193.33 (26.82)	200.76 (44.12)	0.238	197.41 (11.63)	200.34 (49.54)	0.846
CHG−WC *	626.50 (122.73)	652.67 (120.74)	0.249	641.53 (116.31)	652.62 (125.79)	0.872
CHG−WHtR *	3.64 (0.94)	3.95 (0.89)	0.091	3.92 (0.76)	3.87 (0.93)	0.594

IR: insulin resistance; PAD: peripheral artery disease; DPN: diabetic peripheral neuropathy; TyG: triglyceride-glucose; TyG–BMI: TyG–body mass index; TyG–WC: TyG–waist circumference; TyG–WHtR: TyG–waist-to-height ratio; TG/HDL-c: triglyceride to high-density-lipoprotein cholesterol; MetS-IR: metabolic score for insulin resistance; CHG: cholesterol, HDL, glucose; CHG−BMI: CHG−body mass index; CHG−WC: CHG−waist circumference; CHG−WHtR: CHG−waist-to-height ratio. Continuous variables with normal distribution are presented as mean ± standard deviation, and those with abnormal distribution are presented as median (IQR). * Mann–Whitney U test was used for this analysis; ** Student’s *t* test was used for this analysis.

**Table 3:** Inflammation biomarkers in patients with PAD vs. DPN.

Inflammation Biomarkers	PAD			DPN		
	PAD (−)	PAD (+)	*p* Value	DPN (−)	DPN (+)	*p* Value
Ferritin (ng/mL)	207.00 (135.00)	178.00 (101.00)	0.114	170.00 (110.00)	178.50 (155.00)	0.710
ESR (mm/hr)	26.50 (27.00)	34.00 (44.00)	0.640	25.00 (26.00)	34.00 (45.00)	0.560
CRP (mg/L)	0.54 (0.50)	0.54 (0.55)	0.537	0.41 (0.64)	0.60 (0.54)	0.201
Fibrinogen (mg/dL)	333.00 (108.8)	354.50 (147.00)	0.763	336.50 (118.00)	377.00 (194.00)	0.383
TNFα (pg/mL)	69.76 (34.80)	76.24 (51.13)	0.316	69.12 (37.24)	77.03 (30.64)	0.028
IL-6 (pg/mL)	7.29 (6.16)	8.71 (7.10)	0.026	6.90 (3.82)	8.67 (6.71)	0.063
FGF1 (pg/mL)	532.82 (219.44)	574.38 (101.01)	0.121	544.27 (209.59)	565.65 (159.09)	0.238
MMP1 (pg/mL)	1309.67 (788.00)	1541.82 (789.73)	0.602	1371.77 (770.81)	1382.25 (928.17)	0.872
NLR	2.00 (1.00)	2.00 (1.00)	0.729	2.00 (1.00)	2.00 (1.00)	0.220
MLR	0.20 (0.06)	0.22 (0.10)	0.237	0.19 (0.07)	0.22 (0.10)	0.008
SII	357.09 (248.46)	517.11 (252.33)	0.015	380.46 (199.92)	489.53 (266.72)	0.137
CAR	0.11 (0.16)	0.16 (0.14)	0.410	0.08 (0.12)	0.16 (0.16)	0.038
NPAR	1233.00 (318.00)	1293.00 (352.00)	0.056	1190.00 (352.00)	1291.50 (348.00)	0.497

PAD: peripheral artery disease; DPN: diabetic peripheral neuropathy; ESR: erythrocyte sedimentation rate; CRP: C-reactive protein; TNFα: tumor necrosis factor alpha; IL-6: interleukin-6; FGF1: fibroblast growth factor 1; MMP1: matrix metalloproteinase 1; NLR: neutrophil-to-lymphocyte ratio; MLR: monocyte-to-lymphocyte ratio; SII: systemic immune-inflammation index; CAR: C-reactive protein-to-albumin ratio; NPAR: neutrophil percentage-to-albumin ratio. Continuous variables with abnormal distribution are presented as median (IQR). Mann–Whitney U test was used for all these analyses.

**Table 4:** The ROC curve analysis for the IR, inflammation biomarkers, and DM in PAD.

IR and Inflammation Biomarkers	AUROC Curve	Standard Error	95% CI	*p* Value	Cut-Off Value	Sensitivity (%)	Specificity (%)
TyG−WHtR	0.629	0.067	0.498–0.759	0.042	5.78	75.6	50.0
CHG	0.553	0.069	0.417–0.689	0.403	–	–	–
IL-6 (pg/mL)	0.641	0.059	0.526–0.756	0.026	8.39	61.0	71.4
SII	0.655	0.065	0.528–0.782	0.015	334.27	87.8	50.0
DM duration (years)	0.813	0.044	0.726–0.900	<0.001	7.50	70.7	78.6

IR: insulin resistance; ROC: receiver operating characteristic; AUROC: area under the receiver operating characteristic; CI: confidence interval; TyG–WHtR: TyG–waist-to-height ratio; CHG: cholesterol, HDL, glucose; IL-6: interleukin-6; SII: systemic immune-inflammation index; DM: diabetes mellitus.

**Table 5:** The ROC curve analysis for the IR, inflammation biomarkers, and DM in DPN.

IR and Inflammation Biomarkers	AUROC Curve	Standard Error	95% CI	*p* Value	Cut-Off Value	Sensitivity (%)	Specificity (%)
CHG	0.552	0.088	0.379–0.724	0.487	–	–	–
TNFα (pg/mL)	0.664	0.057	0.552–0.776	0.028	75.91	77.8	58.7
MLR	0.697	0.062	0.576–0.818	0.008	80.50	41.3	55.6
CAR	0.655	0.074	0.510–0.800	0.039	0.20	60.9	77.8
DM duration (years)	0.982	0.010	0.962–1.000	<0.001	4.50	91.3	100.0

IR: insulin resistance; ROC: receiver operating characteristic; AUROC: area under the receiver operating characteristic; CI: confidence interval; CHG: cholesterol, HDL, glucose; TNFα: tumor necrosis factor alpha; MLR: monocyte-to-lymphocyte ratio; CAR: C-reactive protein-to-albumin ratio; DM: diabetes mellitus.

**Table 6:** The score associated with PAD and DPN in subjects with type 2 DM.

Items	0 Points	1 Point
DM duration (years)	<4.50	≥4.50
TyG−WHtR	<5.78	≥5.78
SII	<334.27	≥334.27
MLR	<80.50	≥80.50
CAR	<0.20	≥0.20

PAD: peripheral artery disease; DPN: diabetic peripheral neuropathy; DM: diabetes mellitus; TyG–WHtR: TyG–waist-to-height ratio; SII: systemic immune-inflammation index; MLR: monocyte-to-lymphocyte ratio; CAR: C-reactive protein-to-albumin ratio.

**Table 7:** The ROC curve analysis for the proposed score associated with PAD and DPN.

Score Predicting	AUROC Curve	Standard Error	95% CI	*p* Value	Cut-Off Value	Sensitivity (%)	Specificity (%)
PAD	0.822	0.043	0.738–0.907	<0.001	2.50	63.4	85.7
DPN	0.848	0.039	0.772–0.923	<0.001	2.50	60.9	100.0

PAD: peripheral artery disease; DPN: diabetic peripheral neuropathy; ROC: receiver operating characteristic; AUROC: area under the receiver operating characteristic; CI: confidence interval.

**Table 8:** Inflammation biomarkers definition.

Biomarker	Formula	Reference
NLR	neutrophil count (×10^3^ cells/μL)/lymphocyte count (×10^3^ cells/μL)	[21]
MLR	monocyte counts (×10^3^ cells/μL)/lymphocyte counts (×10^3^ cells/μL)	[60]
SII	platelet (×10^3^ cells/μL) × (neutrophil count (×10^3^ cells/μL)/lymphocyte count (×10^3^ cells/μL))	[23]
CAR	C-reactive protein (mg/L)/serum albumin (g/L)	[24]
NPAR	(neutrophil percentage (%) × 100)/serum albumin (g/dL)	[60]

NLR: neutrophil-to-lymphocyte ratio; MLR: monocyte-to-lymphocyte ratio; SII: systemic immune-inflammation index; CAR: C-reactive protein-to-albumin ratio; NPAR: neutrophil percentage-to-albumin ratio.

**Table 9:** Insulin resistance biomarkers definition.

Biomarker	Formula	Reference
TyG	Ln (fasting triglycerides (mg/dL) × fasting glucose (mg/dL)/2)	[28]
TyG−BMI	TyG × BMI (kg/m^2^)	[29]
TyG−WC	TyG × WC	[29]
TyG−WHtR	TyG × WHtR	[30]
TG/HDL-c	fasting triglycerides (mg/dL)/fasting HDL-c (mg/dL)	[61]
MetS-IR	[Ln (2 × fasting glucose (mg/dL) + fasting triglycerides (mg/dL)) × BMI (kg/m^2^)]/[Ln (HDL-c (mg/dL))]	[31]
CHG	Ln [(Total Cholesterol (mg/dL) × fasting glucose (mg/dL))/(2 × HDL-c (mg/dL))]	[62]
CHG−BMI	CHG × BMI (kg/m^2^)	[62]
CHG−WC	CHG × WC	[62]
CHG−WHtR	CHG × WHtR	[26]

TyG: triglyceride-glucose; TyG–BMI: TyG–body mass index; TyG–WC: TyG–waist circumference; TyG–WHtR: TyG–waist-to-height ratio; TG/HDL-c: triglyceride to high-density-lipoprotein cholesterol; MetS-IR: metabolic score for insulin resistance; CHG: cholesterol, HDL, glucose; CHG−BMI: CHG−body mass index; CHG−WC: CHG−waist circumference; CHG−WHtR: CHG−waist-to-height ratio. Ln represents natural logarithm.

## Data Availability

The original contributions presented in this study are included in the article. Further inquiries can be directed to the corresponding authors.

## Abbreviations

The following abbreviations are used in this manuscript:

ABI	ankle–brachial index
AUROC	area under the receiver operating characteristic
BMI	body mass index
CAR	C-reactive protein-to-albumin ratio
CHG	cholesterol, HDL, glucose
CHG−BMI	CHG−body mass index
CHG−WC	CHG−waist circumference
CHG−WHtR	CHG−waist-to-height ratio
CI	confidence interval
CRP	C-reactive protein
DM	diabetes mellitus
DPN	diabetic peripheral neuropathy
ESR	erythrocyte sedimentation rate
FGF1	fibroblast growth factor 1
FPG	fasting plasma glucose
GCP	good clinical practice
HbA1c	glycated hemoglobin
HDL-c	high-density lipoprotein cholesterol
ICH	International Conference on Harmonization
IDF	International Diabetes Federation
IL	interleukin
IL-6	interleukin-6
IQR	interquartile range
IR	insulin resistance
LDL-c	low-density lipoprotein cholesterol
METS-IR	metabolic score for insulin resistance
MLR	monocyte-to-lymphocyte ratio
MMP1	matrix metalloproteinase 1
NLR	neutrophil-to-lymphocyte ratio
NPAR	neutrophil percentage-to-albumin ratio
PAD	peripheral arterial disease
PN	peripheral neuropathy
PREDATORR	Prevalence of diabetes mellitus, prediabetes, overweight, obesity, dyslipidemia, hyperuricemia, and chronic kidney disease in Romania
ROC	receiver operating characteristic
SD	standard deviation
SII	systemic immune-inflammation index
SPSS	Statistical Package for the Social Sciences
STROBE	The Strengthening the Reporting of Observational Studies in Epidemiology
TC	total cholesterol
TG	triglycerides
TG/HDL-c	triglyceride to high-density-lipoprotein cholesterol
TNF-α	tumor necrosis factor alpha
TyG	triglyceride-glucose
TyG−BMI	TyG−body mass index
TyG−WC	TyG−waist circumference
TyG−WHtR	TyG−waist-to-height ratio
WC	waist circumference
WHtR	waist-to-height ratio
